# Supplementary material for: A descriptive study of zoonotic disease risk at the human-wildlife interface in a biodiversity hot spot in South Western Uganda
Source: PLoS Negl Trop Dis. 2021 Jan 6;15(1):e0008633. doi: 10.1371/journal.pntd.0008633 (PMC7845987; doi:10.1371/journal.pntd.0008633)
Supplement: Attachment — Submitted filename: review.docx [file pntd.0008633.s001.docx]

Major comments

Taxonomic Background – Throughout the manuscript, there is a severe lack of background information about the specific mammal species that the authors are referring to. I realize that this was not part of the surveys, and that the authors may not be taxonomic experts, but it is critical to provide this type of background for future researchers (who may also be working on disease outbreaks in proximate locations). What are the most common species of cane rats, other commensal rats, non-human primates, and ruminants that are in this specific area? As written, its hard to know if the most hunted wildlife species are primarily forest-associated, and therefore whether proximity to the forests is enhancing illness risk, or just interactions with wildlife in general that is irrespective of the study region. This is a critical component to include in a revised manuscript.

Places where this information is needed are: Line 39, Lines 242-244, and Figure 2.

Map (Fig 1) – The map needs additional components to make it a cartographically acceptable representation. Please include a country label for Uganda (as already included for DRC). Please remove ‘Wildlife Interaction’ from the title, as the map does not show these interactions specifically (just their physical context). Finally, please label the 2 subcounties in the map that are mentioned in the text (K & K).

Structure of ‘Results’ section – This section is currently very difficult to follow. In the Introduction, the two phases of the study are pitched as ‘Qualitative’ and ‘Quantitative’, but there is no such distinction in the Results. Please make the distinction between the 2 sets of results clearer. In the description of the quantitative survey results, please also remove or reword the subtitle ‘Descriptive summary’ (301), as this detracts from the quantitative nature of the section it describes.

Statistical Analyses – In general, there is insufficient detail about what specific regression models were constructed. Relatedly, in Table 4, there is a lack of detail about what tests these results came from, and no description of the metrics (cOR, 95% CI, AOR, etc). Please add more detail about the analyses you performed, and please define these acronyms in Table 4 more fully. Finally, in Line 345, please describe what is meant by ‘adjusting for confounding’.

Discussion/Interpretation of the zoonosis knowledge base in Uganda – In general I think there are more findings that can be expounded on in the Discussion section. In my view, there are several interesting facets of the ‘human knowledge’ component of the survey results that specifically deserve more attention in the Discussion section.

1) For example, many respondents had not heard of wildlife diseases, but have nevertheless heard of EBOV/Marburg.
2) Also, the authors state that there is a reduced risk of becoming ill for respondents without zoonosis knowledge, but an increased risk for those knowledgeable about EBOV/Marburg.

These findings potentially suggest (but do not prove) that knowledge about zoonoses is not filtering down substantially enough to impact human behaviors regarding wildlife interaction, despite the fact that knowledge about the existence of specific zoonoses (EBOLA) is filtering down. This is an interesting finding because it has implications for zoonotic spread and the extent to which education or dissemination of information can mitigate future outbreaks. I strongly suggest building out a section in the Discussion related to this or similar topic.

Minor grammatical comments

28 - “areas less aware” -> “areas, but may be less aware”

36-38 – these are the perceived benefits, not the confirmed benefits. Please reword to make this clearer.

46 – “fora” -> “for a”

77 – ‘imperilled’-> I suggest a different word here

79 – ‘also rising’ -> ‘but also commonly to’

85 – Please define EBOV here (this is the first use of the acronym)

85 – ‘Hoima, In’ -> ‘Hoima, in’

88 – Please include reference to specific article(s)

93-101 – this section is repeated from Lines 83-92. Please omit one of these text blocks.

106 –‘between, animals’ -> ‘between animals’

116-117 – Please reword for clarity.

125 – ‘and if they do’ -> ‘and, if they do’

135 – ’34,810’ -> what are the units here? Human population?

161 – This sentence regarding structure of focal groups is repeated in the following section (‘Qualitative data collection’). I recommend removing this instance and leaving the latter.

170 – Please reword and include proper subject for the second sentence.

179 – ‘a total of total’ -> ‘a total of’

185 – Consent to participate was already described in a previous section. Please remove this for clarity.

191 – Please describe in more detail the ‘activities’ you are referring to, which are involved in human-wildlife interactions.

192 – Please include reference to the correct Supplement number or element.

212 – ‘Data summarized’ -> ‘Data were summarized’

227 – ’74.4%’ -> does this refer to the total for water, or for use of all resources? Please clarify.

230-231 – The quotes from discussion groups are informative, but when included in the text they are distracting. If this is acceptable to the journal, I recommend moving all quotes (regardless of length) to an indented section.

254 – Please reword the end of this sentence (including ‘Table 1’ in parentheses).

264 – ‘wildlife;’ -> replace with comma

267 – Please define TB (first usage of the acronym in the article)

281-282 – Please reword this sentence for clarity.

312 – ‘edible rats’ -> ‘hunted edible rats’

314 – Please place 4.5% in parentheses

408-409 – ‘encroachment background’ -> reword for clarity

414 – Revise punctuation in this sentence for clarity

431 – ‘Zoonoses endemic areas’ -> ‘zoonosis hotspots’

Comments on Figures and Tables

Table 2:

- I recommend moving the Location variable (sub-county) to the top of this table, because this is an important descriptor of the dataset (relative to another variable potentially).
- For the ‘House Type’ variable, what is meant by ‘Temporally’ and ‘Semi-permanent’? The first is unclear (maybe this means renting), and the second is also unclear (but I dont know what it might refer to other than the construction quality of houses).

Table 3:

- Please reword the last part of the table caption (including the dates).

Figure 2:

- This figure is a nice addition to the paper. However, the dichotomy between Direct and Indirect Pathways is in some ways a false one, at least as depicted. The consumption of bushmeat and the medicinal use of bushmeat/parts both occur through handling of wildlife; therefore, they both represent direct modes of transmission. Please provide a better distinction between these activities or combine them under Direct modes. Conversely, sharing of water seems to me to be an indirect pathway. Please move this to ‘Indirect Pathways’ or describe why you consider this to be a direct pathway. Lastly, what does the phrase ‘Multiple factors at play’ mean as an outcome? Please clarify this as well.
