# Supplementary material for: A descriptive study of zoonotic disease risk at the human-wildlife interface in a biodiversity hot spot in South Western Uganda
Source: PLoS Negl Trop Dis. 2021 Jan 6;15(1):e0008633. doi: 10.1371/journal.pntd.0008633 (PMC7845987; doi:10.1371/journal.pntd.0008633)
Supplement: Attachment — Submitted filename: review2.docx [file pntd.0008633.s002.docx]

Results/Qualitative : When describing the results, I think it would be helpful if the section headings were identical to what was mentioned in the Methods section as having been collected. Please work to make the variables and their names consistent throughout.

Table 2 : I was still confused about which are the independent and dependent variables (or, the treatment and response). I could not understand this from the Methods text, and I also did not understand it from the Table here. In all tests, I think either location or response (sick Y/N) is present? Please clarify what data the Chi-squared tests were performed on.

Figure 2 : I appreciate the author’s revisions of this figure. I have a few more comments that may further increase readability. First, I suggest including a title for the middle box that reads ‘Livelihood activities that increase risk’. Then. I suggest listing the livelihood activities in bulleted list, not as a sentence as currently done.

81 – ‘Its’ -> ‘It is’

87-89 – I recommend moving this sentence introducing Uganda to the beginning of second paragraph

94 – ‘an’ -> ‘and’

96 -> ‘ins’ -> ‘in’

98 – Please change the specific epithet to lower-case.

105 – Please remove dash in genus name.

113 – Please move abbreviation ‘EBOV’ up to the first usage of the term.

125 – ‘of’ -> ‘or’

134-137 – If you choose to enumerate the main points of the study, please use a) – d)

169-170 – I am not sure what this sentence corresponds to. Please place it in context or remove.

179 – ‘excel’ -> ‘Microsoft Excel’ and please add software version

181 – ‘men and women separate’ -> ‘consisting of 6-10 men or women’

182 – ‘use forest’ -> ‘use of’

195 – Please omit extra quotation

215 – Please re-word the first sentence for clarity.

218-220 – This sentence lacks clarity and its not clear what information was being collected – please re-word.

221 – ‘word’ -> ‘Microsoft Word’

238 – Please re-word this ‘outcome variable’ -> ‘dependent variable’

241 – Please also list the independent variables (again, if necessary) to increase clarity.

245 – What is the metric of model fit that was used? Also, was there a specific function that was used for the stepwise model selection or was it done manually?

258 – Does this percentage refer just to use of animals? Or to the combined use of all three of the things listed?

284/Table – I think that these three entries are just representative examples, correct? Either way, please modify the table caption so it is clear why the table contains these specific entries.

336 – Only >50% or more of responses would be categorized as ‘most’. Please re-word.

350 – Similar to my comment above – does this percentage refer to the combined totals of both these groups, or just the latter?

359 – Table 2 caption – ‘consumption bushmeat’ -> ‘consumption of bushmeat’

430 – ‘Well as studies have shown’ –> ‘Studies have also shown’

440-441 – I am not clear on the meaning of this last sentence. Please reword for clarity.

451-453 – I think this sentence is slightly repetitive with previous text, and I recommend omitting.
