# Supplementary material for: A descriptive study of zoonotic disease risk at the human-wildlife interface in a biodiversity hot spot in South Western Uganda
Source: PLoS Negl Trop Dis. 2021 Jan 6;15(1):e0008633. doi: 10.1371/journal.pntd.0008633 (PMC7845987; doi:10.1371/journal.pntd.0008633)
Supplement: Attachment — Submitted filename: Review 2 cover letter.docx [file pntd.0008633.s003.docx]

July 18, 2020

Shamilah Namusisi

Corresponding Author

shamilahnamusisi@gmail.com

To Editor

PLOS Neglected Tropical Diseases

**RE: Response to second review comments and submission of revised manuscript PNTD-D-19-00311**

# Following submission of manuscript titled “A descriptive study of zoonotic disease risk at the human-wildlife interface in a biodiversity hot spot in South Western Uganda”, below is the response to review two comments

Results/Qualitative: When describing the results, I think it would be helpful if the section headings were identical to what was mentioned in the Methods section as having been collected. Please work to make the variables and their names consistent throughout.

**Response:** This has been aligned

Table 2: I was still confused about which the independent are and dependent variables (or, the treatment and response). I could not understand this from the Methods text, and I also did not understand it from the Table here. In all tests, I think either location or response (sick Y/N) is present? Please clarify what data the Chi-squared tests were performed on.

**Response:** This has been revised

Figure 2: I appreciate the author’s revisions of this figure. I have a few more comments that may further increase readability. First, I suggest including a title for the middle box that reads ‘Livelihood activities that increase risk’. Then. I suggest listing the livelihood activities in bulleted list, not as a sentence as currently done.

**Response:** Figure 2 edited as suggested

81 – ‘Its’ -> ‘It is’,

**Response:** changed to ‘It is’

87-89 – I recommend moving this sentence introducing Uganda to the beginning of second paragraph

**Response:** line 88-89 ‘ beginning of the second paragraph ‘Uganda’s vulnerability to climate change has been highlighted and is .bound to increase because many livelihood are dependent on natural resources’

94 – ‘an’ -> ‘and’

**Response:** d added to read and

96 -> ‘ins’ -> ‘in’

**Response:** s removed

98 – Please change the specific epithet to lower-case.

**Response:** line 97 changed to lover case Changed

105 – Please remove dash in genus name.

**Response**: dash removed

113 – Please move abbreviation ‘EBOV’ up to the first usage of the term.

**Response**: line 111: This is where is was first mentioned

125 – ‘of’ -> ‘or’

**Response**: of deleted for the sentence to read ‘Human activities like encroachment on wildlife habitat for agriculture purpose’

134-137 – If you choose to enumerate the main points of the study, please use a) – d)

**Response**: Line 132-135 : used the a)- d) to highlight the focus of the study.

169-170 – I am not sure what this sentence corresponds to. Please place it in context or remove. **Response**: Line 168-169: The sentence corresponds to the project source of the map

179 – ‘excel’ -> ‘Microsoft Excel’ and please add software version

**Response**: line 178-179: changed to ‘Microsoft Excel version 2016’

181 – ‘men and women separate’ -> ‘consisting of 6-10 men or women’

**Response**: line 180: Clanged to ‘consisting of 6-10 men or women’

182 – ‘use forest’ -> ‘use of’

**Response**: line 181: changed to ‘use of’

195 – Please omit extra quotation

**Response**: line 196: removed the extra quotation that appeared on UNCST

215 – Please re-word the first sentence for clarity.

**Response**: LINE 219-222 changed

218-220 – This sentence lacks clarity and it’s not clear what information was being collected – please re-word.

**Response**: line 212-215 changed for clarity

221 – ‘word’ -> ‘Microsoft Word’

**Response:** line 225: changed to ‘Microsoft Word’

238 – Please re-word this ‘outcome variable’ -> ‘dependent variable’

Response: line 238 and 244- changed to ‘dependent variable’

241 – Please also list the independent variables (again, if necessary) to increase clarity.

Response: The independent variables included Sex, age groups, household size, knowledge of diseases transmitted by wild animals to human, ever heard of Ebola or Marburg, mostly frequently hunted animals and Number of people involved in preparation of hunted animals.

245 – What is the metric of model fit that was used? Also, was there a specific function that was used for the stepwise model selection or was it done manually?

**Response:** line 251-252: ‘The model fit was assessed using log-likelihood chi-square and the stepwise model selection was done manually’.

258 – Does this percentage refer just to use of animals? Or to the combined use of all three of the things listed?

**Response:** Line 261-262: changed for clarity

284/Table – I think that these three entries are just representative examples, correct? Either way, please modify the table caption so it is clear why the table contains these specific entries.

**Response: line 293** headline changed to show that these are representative examples on how the communities use wildlife and or their parts

336 – Only >50% or more of responses would be categorized as ‘most’. Please re-word.

**Response:** line 345: ‘Most’ deleted

350 – Similar to my comment above – does this percentage refer to the combined totals of both these groups, and just the latter?

**Response**: line 358: ‘22.7%, CI: 18.6-27.4’ is combined for both cultural practices and medicinal use

359 – Table 2 caption – ‘consumption bushmeat’ -> ‘consumption of bushmeat’

**Response:** line 367: of added to read ‘consumption of bushmeat

430 – ‘Well as studies have shown’ –> ‘Studies have also shown’

**Response: line 431:** ‘Studies have also shown

440-441 – I am not clear on the meaning of this last sentence. Please reword for clarity.

**Response: line 444-445 ‘**For example, 63% of respondents did not understand that Ebola or Marburg is associated with people coming into close contact with wildlife like Chimpanzees’.

451-453 – I think this sentence is slightly repetitive with previous text, and I recommend omitting.

**Response:** sentence deleted at line 455
